# An Improved Lagrangian-Inverse Method for Evaluating the Dynamic Constitutive Parameters of Concrete

**DOI:** 10.3390/ma13081871

**Published:** 2020-04-16

**Authors:** Xinlu Yu, Yingqian Fu, Xinlong Dong, Fenghua Zhou, Jianguo Ning

**Affiliations:** 1 School of Mechatronical Engineering, Beijing Institute of Technology, Beijing 100081, China; 2 Key Laboratory of Impact and Safety Engineering (Ningbo University), Ministry of Education, Ningbo 315211, China

**Keywords:** concrete-like materials, dynamic constitutive behaviors, lagrangian-inverse analysis, Digital image correlation, regularization, non-liner elastic constitution

## Abstract

The dynamic constitutive behaviors of concrete-like materials are of vital importance for structure designing under impact loading conditions. This study proposes a new method to evaluate the constitutive behaviors of ordinary concrete at high strain rates. The proposed method combines the Lagrangian-inverse analysis method with optical techniques (ultra-high-speed camera and digital image correlation techniques). The proposed method is validated against finite-element simulation. Spalling tests were conducted on concretes where optical techniques were employed to obtain the high-frequency spatial and temporal displacement data. We then obtained stress–strain curves of concrete by applying the proposed method on the results of spalling tests. The results show non-linear constitutive behaviors in these stress–strain curves. These non-linear constitutive behaviors can be possibly explained by local heterogeneity of concrete. The proposed method provides an alternative mean to access the dynamic constitutive behaviors which can help future structure designing of concrete-like materials.

## 1. Introduction

Concrete-like brittle materials (such as cement, mortar, concrete, and geomaterials) are widely used in civil and military structural engineering [1,2,3]. Recently, it becomes a growing trend to improve the impact resistance of structures with these concrete-like brittle materials [4,5,6]. Evaluating the effective constitutive parameters of these materials under dynamic condition is thus of vital importance for designing the structures [7,8,9,10].

The constitution relation, which describes the material behavior, is generally presumed to be the functions. Parameters in these functions are mainly obtained by the curve fitting to the experimental data. Among those of constitutive models, the linear-elastic model is the simplest and most widely used one [11]. In the linear-elastic model, concrete is treated as linear-elastic until it reaches ultimate strength and subsequently it fails in brittle manner. However, non-linear elastic behaviors were reported when the concrete under multi-axial stress states or high-rate loading conditions [11]. Especially for the high-rate loading conditions, the multiple-phase components in concrete lead to the occurrence of chaos during the process of wave propagation. The uncertainty in the constitutive behavior of these materials may then yield significant errors in the estimated constitutive parameters. It thus becomes necessary to develop a dynamic test technique which does not require any presumed constitutive relations for concrete.

The inertia effect and strain-rate effect are generally required to be considered for evaluating the dynamic responses of materials under impact loading condition [12,13,14,15]. However, they are generally inter-coupled in the dynamic tests. To solve such coupled problem, two techniques are commonly used to investigate the dynamic behavior of materials: the split Hopkinson pressure bar technique (SHPB) [16,17] and the wave propagation technique (WPT) [14].

The SHPB and Kolsky bar technique are widely used to conduct the test of dynamic constitutive behaviors on concrete-like materials [18,19,20]. However, the validity of SHPB results highly depends on the assumptions of one-dimensional stress wave propagation and stress uniformity along the specimen length [14]. To get the average mechanical properties of multiple-phase materials, the diameters of specimen and pressure bar should be at least five times larger than the mean diameter of coarse aggregates in the SHPB test for concrete materials. However, the transverse inertia effect can be significant in the bars with a large diameter. Also, it is challenging to reach a non-uniform distribution of stress/strain along the specimen length for a relatively large-size concrete specimen under high strain-rate loading conditions.

The wave propagation technique (WPT) implicitly considers the interactions between waves and strain-rate effects [21]. We can directly obtain the rate-dependent constitutive relation from a series of wave propagation measurements, which is generally called “second class of inverse problem mathematically”. The Lagrangian analysis is an attractive inverse method because it does not require any presumed constitution relations [22,23,24,25,26,27]. This method has been effectively applied to investigate the high-strain-rate constitutive response of concrete-like materials [28,29]. However, one challenge of this method is that the boundary stress and particle velocity should be simultaneously measured at one position by two gauges [21,30].

Wang et al. [21] proposed a method which combines the Lagrangian analysis and SHPB technique. The authors named this method as “1sv + nv” or “1sε + nε” inverse analysis method This method has been successfully applied on polymer and cellular materials [15,21,31]. However, limited research has been reported to apply this method to evaluate the dynamic constitutive parameters of concrete-like materials. In the previous literature of Lagrangian analysis [15,21,32], limited number of strain or velocity gauges is employed in traditional measurement. The relatively low spatial and temporal resolution in traditional measurements significantly restricts the wide application of Lagrangian analysis for evaluating the dynamic constitutive behaviors of concrete-like materials.

The recent developments in optical techniques (ultra-high-speed camera and digital image correlation) make it possible to obtain high-resolution and -frequency data, which can remarkably improve the application of inverse analysis methods on high strain-rate experiments. Studies [33,34,35] have employed these techniques for spalling tests based on the virtual field method (VFM) [36,37,38]. These studies demonstrate that these techniques can effectively characterize the tensile strength, Young’s modulus, the stress–strain response, and the fracture energy of brittle materials at high strain rate. It is thus of great interest to combine these techniques with the Lagrangian analysis method for evaluating the constitutive behaviors of concrete-like materials at high strain-rate loading tests.

This study aims to evaluate the dynamic constitutive behaviors of ordinary concrete at high strain rates through combing Lagrangian analysis method with optical techniques. We propose an improved “1sv + nv” method [15,21], and then validate it against finite-element simulation. We further apply the proposed method on the spalling tests to evaluate the constitutive behaviors of ordinary concrete at high strain rates.

In Section 2, we report the basic theories of Lagrangian analysis method and our improved “1sv + nv” method applied in spalling test. We then describe the simulation model and experimental design for the spalling test in details. In the experimental program, we introduce the materials and specimen, setups and data acquisitions, and the testing procedures. The simulated and experimental results are then shown in Section 3. We validate the proposed method against the simulation results, and then apply the proposed method on the experimental results to evaluate the dynamic constitutive behaviors of concrete. In Section 4, we discuss the non-linear constitutive behaviors of concrete conducted from the experimental results. The contributions and limitations of this work are also discussed in the end.

## 2. Methodology

Overall, this study was conducted via the following four key steps: (1) Develop the improved Lagranagian-inverse analysis method; (2) Conduct finite-element (FE) simulation to validate the proposed method; (3) Conduct spalling tests on concretes to evaluate the constitutive behaviors of concrete at high strain rates. In the spalling tests, high-resolution displacement fields were obtained by the techniques of ultra-high-speed camera and digital image correlation (DIC); (4) Apply the improved Lagranagian analysis method to construct the stress–strain curves for the spalling tests.

### 2.1. Theories

This subsection provides an overview of Lagrangian analysis method, and then describes how the method is modified for spalling tests.

#### 2.1.1. Lagrangian Analysis Method

##### Basic Equations

Considering one-dimensional stress waves propagating in a rate-dependent material with the density of $\rho_{0}$, the momentum conservation and continuity equations can be described by

(1) $$\rho_{0}\frac{\partial v}{\partial t}=\frac{\partial \sigma }{\partial X}$$

(2) $$\frac{\partial v}{\partial X}=\frac{\partial \varepsilon }{\partial t}$$

where Equation (1) describes the relationship between the partial derivative of stress (σ) and the partial derivative of particle velocity (*v*). Similarly, Equation (2) describes the relationship between *v* and the partial derivative of strain (ε). The relationship between σ and ε can then be established through the particle velocity of *v*. Equations (1) and (2) are the theoretical basis for all-kind Lagrange inverse analysis methods used in WPT methods. Inevitably, initial and boundary conditions are required to solve these equations.

##### ”1sv + nv” Method

Wang et al. [21] proposed the “1sv + nv” method which combines the SHPB technique with Lagrangian analysis. In their method, the SHPB incident bar transfers loading to the specimen. More importantly, the SHPB incident bar does not only play a role of transferring loading to the specimen, but also provide a “stress-particle velocity” combining gauge at the interface of bar and specimen. Hence, the stress wave and the particle velocity wave are simultaneously measured at the boundary. *n* profiles of particle velocity wave can then be measured by *n* gauges mounted at *n* Lagrangian positions. σ and ε can further be numerically calculated by the Lagrangian-inverse method using Equations (1) and (2).

##### Path-line Method

High-resolution and -frequency data are generally challenging to be obtained in the traditional experiments. The Lagrangian particles are relatively too sparse to obtain high-accuracy partial derivatives. To overcome this challenge, the path-line method was proposed by assuming that stress wave propagates along a mathematical path line between the two adjacent Lagrangian positions [21,24]. The path-line method then switches the first-order derivatives containing variable *X* to the partial derivatives containing variable *t* by the total differentiation along the path line. The stress–strain curves can then be numerically calculated by the Lagrangian analysis method.

#### 2.1.2. Improved “1sv + nv” Method for the Spalling Tests

##### Spalling Tests

**Figure 1:**
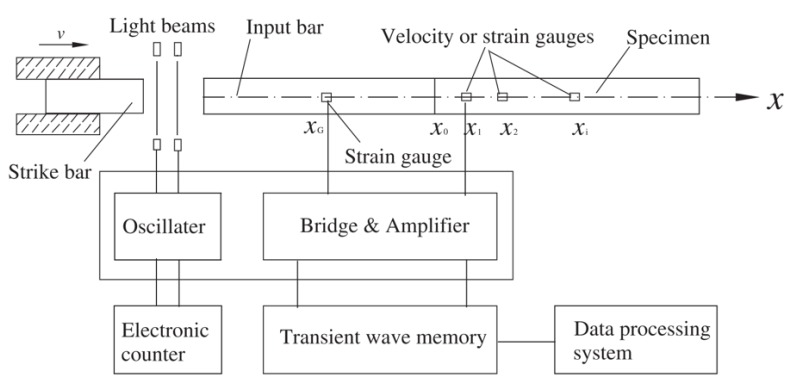
Schematics illustrating Wang et al. [21]’s method which combines Lagrangian analysis with Hopkinson pressure bar technique.

Spalling test has been proved to be an effective method to characterize the dynamic tension behaviors of brittle materials [39,40,41,42,43]. Klepaczko and Brara [39] developed the spalling test on the base of SHPB technique. As shown in Figure 1, their test setup consists of a striking bar, a Hopkinson bar (as the measuring tool), and a relatively long concrete specimen contacting with the incident bar. The incident compression wave transmitted by the Hopkinson bar into the specimen is reflected as a tensile wave, which causes the spalling. Schuler et al. [40] adapted the Novikov-formula method [44] to determine the spalling tensile strength of concrete under 1D-stress condition. In their spalling test, they measured the pull-back velocity at the free end of sample to determine the tensile strength ($\sigma_{fdyn}$) by

(3) $$\sigma_{fdyn}=\frac{1}{2}\rho_{0}C_{0}\Delta V_{pb}$$

where $\rho_{0}$ is the density of concrete; $C_{0}$ is the propagating speed of one-dimensional elastic wave in concrete; and $\Delta V_{pb}$ is the “pull-back” velocity at the free end of specimen. Erzar and Forquin [45] further suggest that the spalling test method using $V_{pb}$ can provide a relatively high accurate tensile strength compared with previous methods.

Linear-elastic constitution relation is commonly presumed in the spalling test method to determine the tensile strength for materials. However, The constitution behaviors generally can be non-linear or inelastic for the quasi-brittle materials such as concrete. The high uncertainty in the constitutive behavior of these materials may thus yield significant errors in the estimated tensile strength. Therefore, it becomes necessary to develop a dynamic test technique which does not require any presumed constitutive relations.

##### Improved “1sv + nv” Method

The Lagrangian-inverse analysis method provides an alternative to evaluate the dynamic stress–strain response and tensile strength without assuming any constitutive relations. However, in Wang et al.’s “1sv + nv” method, gauges are placed at the loading end of concrete bar to constrain the boundary conditions (see Figure 1). Under relatively high strain-rate conditions, the “1sv + nv” method is then only applicable to evaluate the compressive (but not tensile) behaviors of concrete. Hence, the “1sv + nv” method should be further modified to obtain both compressive and tensile responses of concrete under dynamic condition.

In this study, we place a laser velocimetry at the free end of concrete bar to obtain the dynamic tensile response. The reflected tension wave is expected to form at the boundary, and then propagates towards the left end after the wave superposition.

Here, we describe the general procedures of improved “1sv + nv” method as follows:

First, set the stress and velocity of each particle as zero at the initial condition:

(4) $$\left\{\begin{array}{c} \sigma (X,0)=0\\ \varepsilon (X,0)=0 \end{array}\right.$$

Second, select the free end of concrete bar as the boundary. The boundary conditions are then described by

(5) $$\left\{\begin{array}{c} \sigma (0,t)=0\\ v\left(0,t\right)=V_{0}\left(t\right) \end{array}\right.$$

where the boundary particle velocity ($V_{0}$) is the result of wave superposition at the free end of concrete bar, and $V_{0}$ can be measured by a laser velocimetry. As the stress and velocity obey the momentum conservation equation (Equation (1), the zero-stress boundary condition is applied for the improved “1sv + nv” method.

Third, solve the Lagrangian-inverse analysis by combining the path-line and “1sv + nv” methods. However, the path-line method is mathematically assumed for the indeterminacy of stress wave propagation characteristics in a specimen. In fact, the path-line method is unnecessary if high-resolution and -frequency data are available [31]. The high-resolution particle-displacement field (*u*) can be obtained by combining the ultra-high-speed camera with the DIC techniques. The stress and strain fields (ε and σ) can then be determined directly from Equation (1) and Equation (2) in the differential formats of *u* by the inverse method.

The momentum conservation equation and continuity equation with respect to *u* can then be described in the discrete form by

(6) $$\sigma_{i+1,j}-\sigma_{i,j}=\rho_{0}{\left.\frac{\partial^{2}u_{i,j}}{\partial t^{2}}\right|}_{X_{i}}\left(X_{i+1}-X_{i}\right),i=0,1,\cdots ,N-1,j=0,1,\cdots M-1$$

(7) $$\varepsilon_{i,j+1}-\varepsilon_{i,j}={\left.\frac{\partial^{2}u_{i,j}}{\partial X\partial t}\right|}_{t_{j}}\left(t_{j+1}-t_{j}\right),i=0,1,\cdots ,N-1,j=0,1,\cdots M-1$$

where *i* represents the space increment of *X* in Lagrangian coordination; and *j* represents the time increment of *t*.

Studies suggest that the inverse analysis method can be ill-conditioned [46,47,48]. A small change in $\frac{\partial^{2}u}{\partial t^{2}}$ or $\frac{\partial^{2}u}{\partial X\partial t}$ may have a negligible effect on the solution *u*. If it was not carefully examined, however, a minor error in *u* can lead to significant errors in the computed $\frac{\partial^{2}u}{\partial t^{2}}$ and $\frac{\partial^{2}u}{\partial X\partial t}$. A well allegorical method is thus required to reduce the errors in *u* for the effective reconstruction of derivatives.

##### Tikhonov Regularization Method

**Figure A1:**
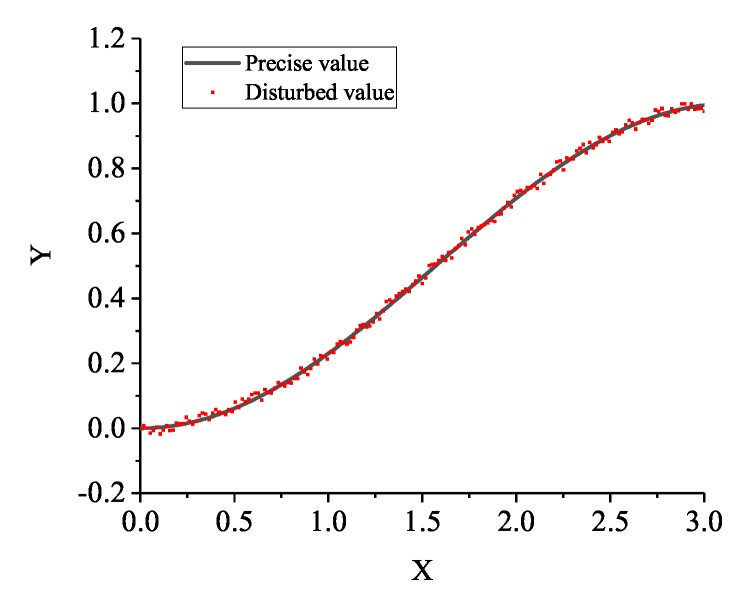
Solid line: $y1=0.5\times \left(1-\cos(x)\right)$, Dot line: y=y1+0.04∗random(−1,1).

**Figure A2:**
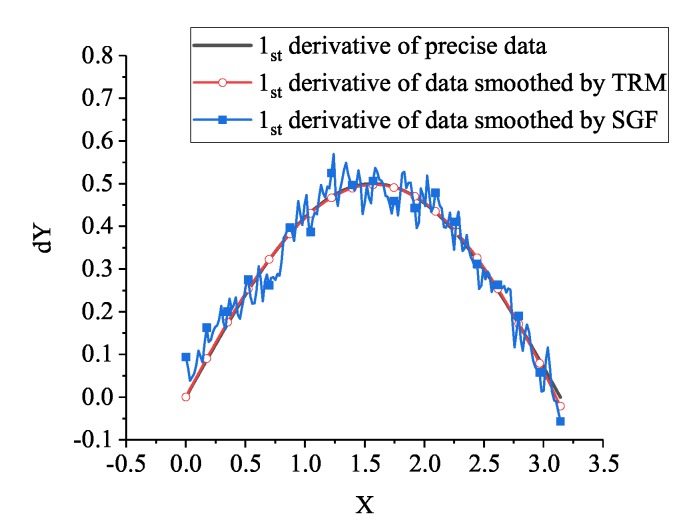
Comparing the first derivative of data smoothed by Tikhonov regulation method (TRM, red line) and Sativetzky–Golay filtering method (SGF, blue line) with $y_{1}^{\prime }$ function (black line).

**Figure A3:**
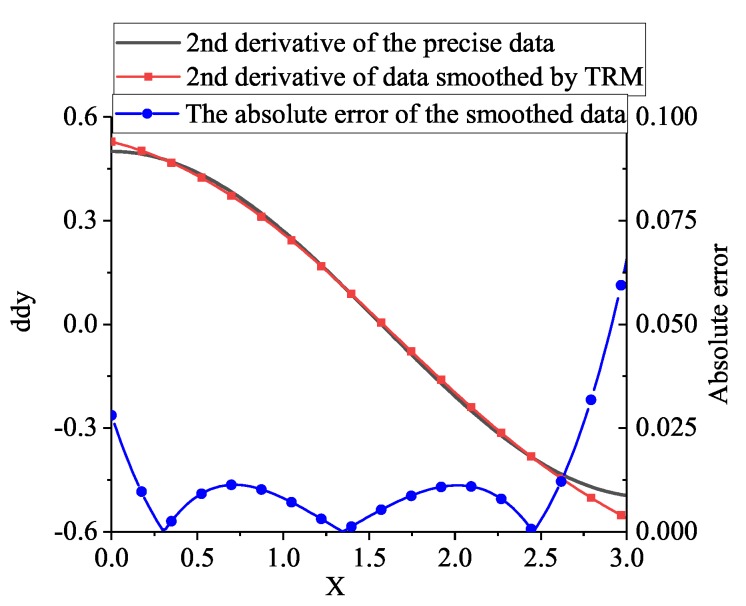
Compare the $2_{st}$ derivative of data between: Solid line: Precise value and red dash dot line: Smoothed by Tikhonov regulation method.

Studies [46,47,48] suggest that the Tikhonov regularization technique can be effectively applied in the inverse analysis method. Wang et al. [48] extended the Tikhonov regularization method to reconstruct high order derivatives (Appendix A describes the detailed algorithms of this method, where shows the basic equations from Equations (A1)–(A5) and a successful case using this method to reconstruct the derivatives as shown in Figure A1, Figure A2 and Figure A3). Similarly, this study adopts the Tikhonov regularization method to reconstruct $\frac{\partial u}{\partial t}$ and $\frac{\partial^{2}u}{\partial t^{2}}$. The difference is that our study obtains the discrete input data of *u* by the DIC method to increase the accuracy in outputs.

### 2.2. Finite-Element Model

One benefit of FE method is that it can provide a complete field of particle velocity, which cannot be fully obtained in experiments. The strain and stress fields in the FE simulation can also be determined directly by the Lagrangian method. In this study, we validated our improved “1sv + nv” method for spalling test against FE simulation. We conducted the FE simulation using the commercial FE software Abaqus/Explicit. This subsection describes the detailed information about the geometry and elements, the constitution of materials, and loading and boundary conditions in the FE model.

#### 2.2.1. Geometry and Element

**Figure 2:**
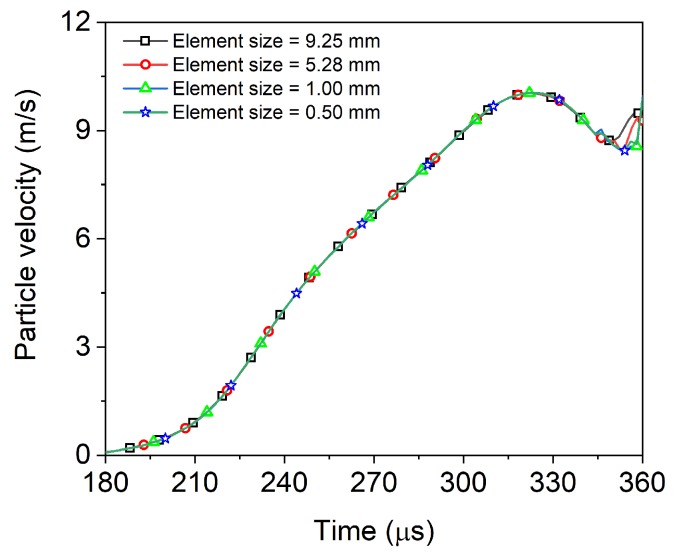
Comparison of particle velocities for FE models with different mesh sizes (0.5 to 9.25 mm) demonstrating that the mesh size of 1 mm is small enough to meet the convergence of simulation.

The single concrete bar in the FE model has the geometrical dimensions of 74 mm in diameter and 1000 mm in length. As shown in Figure 2, the mesh size of concrete bar was set to be 1 mm which is small enough to meet the convergence of simulation.

#### 2.2.2. Material Model

Here, we use an elastic material model of concrete in FE model with a brittle failure constitution based on the principle of maximum tensile stress. A homogeneous and isotropic elastic modulus is set to be 30 GPa with the Poisson ratio of 0.2. The maximum tensile failure stress is assumed to be 6.6 MPa. In this material model, we only considered the tensile damage softening with fracture energy of 140 N/m. The strain-rate effect was not considered here in the simulation.

#### 2.2.3. Loading and Boundary Conditions

**Figure 3:**
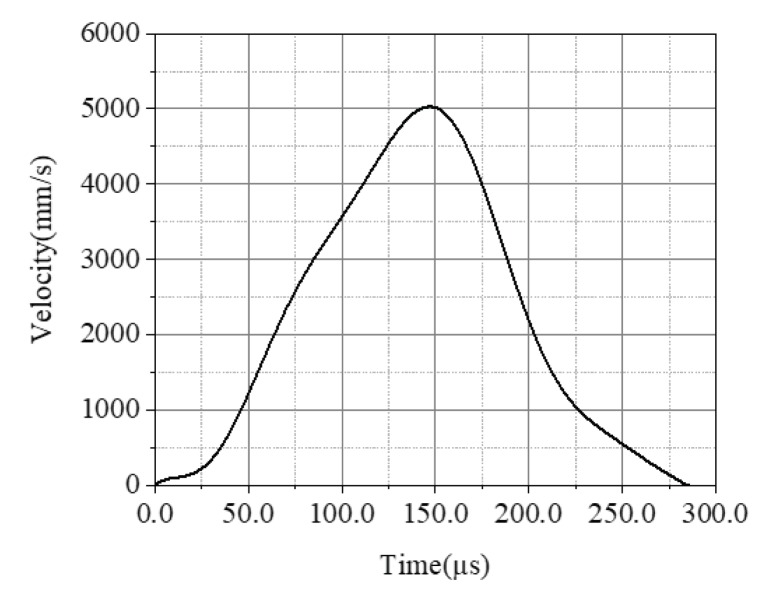
The boundary particle velocity at the loading end of concrete bar.

The boundary stress and particle velocity are coupled at the loading end of concrete bar. To validate the proposed “1sv + nv” method, we set the loading condition in the FE model as a boundary particle velocity shown in Figure 3.

### 2.3. Experimental Program

This subsection describes materials and specimens, setups and data acquisition, and testing procedures for the spalling experiments.

#### 2.3.1. Materials and Specimens

To obtain the constitutive parameters of materials under uniaxial stress state, the geometry of specimen must be a slender bar or sheet. We thus conducted the spalling tests on a relatively long cylindrical concrete bar. However, concrete is one multiple-phase material which includes the cement, fine aggregates, coarse aggregates, and bubbles. Hence, the geometrical dimension is expected to be large enough to obtain the average mechanical properties of the multiple-phase materials. The dimension of specimen is generally required to be 5 times larger than the maximum diameter of coarse aggregates for the ordinary concrete. In this work, the maximum diameter of coarse aggregates is about 12 mm. Our concrete bar thus should be larger than 60 mm in diameter. Meanwhile, the large specimen needs the loading instrument of SHPB with a relatively large diameter. Actually, the bars of SHPB with 74 mm in diameter are the commonly used to conduct the dynamic tests on concrete. Therefore, this study employs a slender bar with a length of 1000 mm, a diameter of 74 mm, and a flatness tolerance of 0.075 mm at both ends for the specimen of spalling tests.

The ordinary Portland cement (P. I. 42.5) with a 28-day compression strength of 42.5 MPa were used. The coarse aggregates are rounded in shape with a size ranging from 12 mm down to 5 mm. The fine aggregates are constituted of river sand with a specific gravity of 2.6. The ratio of water to cement is 0.5.

The mixes were prepared and cured under laboratory conditions. All the samples were then cast simultaneously using the same concrete batches. Afterward, these samples were cured for 28 days under same ambient conditions (20 ± 2 ${}^{\circ }$C and 95% relative humidity). The quasi-static mechanical properties of concrete are summarized in Table 1.

#### 2.3.2. Setups and Data Acquisition

**Figure 4:**
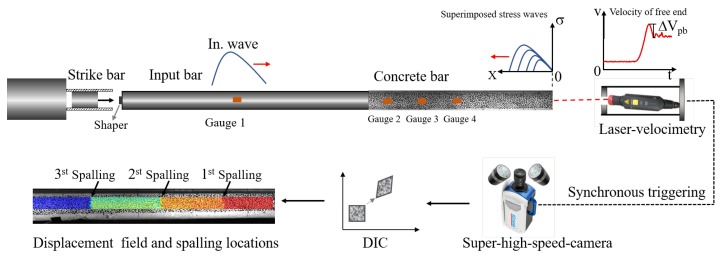
Schematics illustrating the spalling tests combining with the technique of ultra-high-speed camera and DIC method.

**Figure 5:**
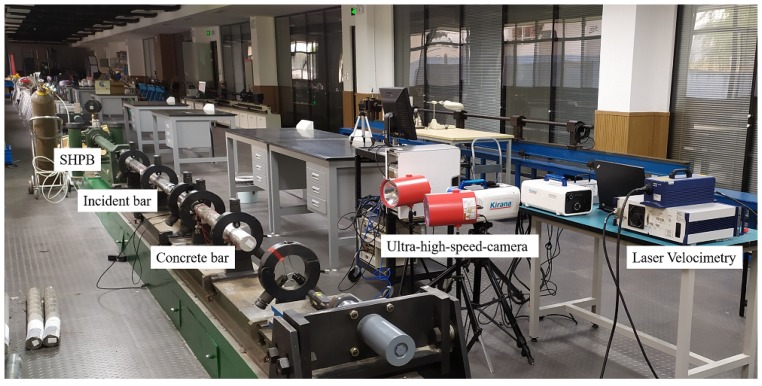
Experiment setups for the spalling tests including the SHPB, the concrete bar, the ultra-high-speed camera, the laser velocimetry, and the data acquisition systems.

As shown in Figure 4, this study conducted the spalling tests using a well-designed setup to evaluate the dynamic tensile behaviors of concrete materials at high strain rates. We employed a Hopkinson input bar with the length of 2.4 m and the diameter of 74 mm to generate a compressive wave and transfer the loading to the concrete specimen. To obtain a range of strain rates, several pure copper ring shapers [49,50,51] were sandwiched between the short strike bar and the input bar to generate the triangle waves with different up-rising slopes. Figure 5 shows the experimental setups including the SHPB, concrete bar, ultra-high-speed camera, laser velocimetry, and data acquisition systems.

As the concrete specimen contacts with the Hopkinson bar, a portion of the incident wave in the input bar is transmitted to the sample whereas, the other portion is reflected to the input bar in the opposite direction. To obtain the profiles of transmitted wave and the propagating performance in concrete, we mounted three strain gauges (Gauge 2, 3, and 4) at the places 100 mm, 300 mm, and 500 mm away from the left end of specimen (see Figure 4).

The compressive transmitted wave will be reflected to be a tensile reflected wave once it arrives at the free end of concrete bar. As shown in Figure 4, the transmitted and reflected waves will then be superimposed to be a growing tensile wave, which propagates to the left end of concrete bar. The spalling failure occurs when the superimposed stress of tensile reflected wave becomes higher than the tensile strength of concrete.

Although the strain-rate effect is one key constitutive behavior, we obtain the strain rates only after the test finished. Hence, it is difficult to control the strain rate before the test. Also, the strain rate of spalling test depends on the magnitude of transmitted wave. However, the transmitted wave is decided by the incident wave which is strongly related to the impact velocity of striker bar. The strain rate can thus be effectively controlled by changing the impact velocity of strike bar. To describe the loading magnitude of spalling tests, we define this impact velocity ($V_{I}$) of striker bar as the loading rate of spalling test. In this article, we employed an optoelectronic velocimetry (a pattern of SHPB system) to measure the impact velocity of striker bar. The loading rates (impact velocities) are listed in Table 2.

The Novikov-formula method requires a pull-back velocity at the free end of concrete bar for calculating the spalling tensile strength as Equation (3). In this study, we adopted a laser velocimetry to measure the particle velocity at the free end ranging from 0 to 100 m/s at the sampling rate of 10 MHz. The laser velocimetry was adopted based on the Laser Doppler Principle.

Obtaining high-resolution and -frequency input data is necessary to accurately reconstruct the derivatives of $\frac{\partial u}{\partial t}$ and $\frac{\partial^{2}u}{\partial t^{2}}$. In this study, we employed an ultra-high-speed camera from Specialized Imaging with a relatively high spatial resolution of 924 × 768 pixels at a frequency of 0.5 Mfps acquiring 180 frames in total. We then digitized the images recorded by ultra-high-speed camera by DIC software with the approximate spatial resolution of less than 0.5 mm for each pixel length after a pre-treatment of noise-deduction. Finally, we obtained the particle-displacement field in the Lagrangian coordination by combining the optical technique of ultra-high-speed camera with DIC method. Additionally, a wide used commercial DIC software, MatchID, developed by the University of Leuven-Technology, is employed to pretreat the noise and calculate the Lagrangian particle variables.

Additionally, the ultra-high-speed camera and laser velocimetry were triggered synchronous by the same voltage pulse from the strain gauge (Gauge 1) mounted at the input bar. Meanwhile, a super-dynamic data acquisition system was used to record the data synchronously with a sampling rate of 1 MHz in each channel.

#### 2.3.3. Testing Procedures

**Figure 6:**
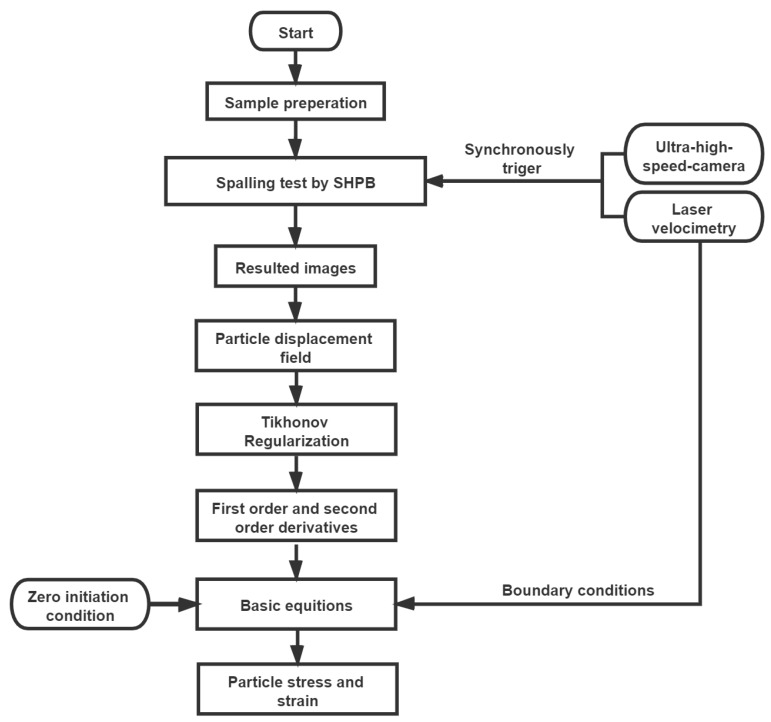
The flowchart of testing procedures.

Figure 6 shows the flowchart which illustrates the following three key testing procedures:

(1) Prepare 9 specimens for the spalling tests under different strain rates;
(2) Conduct the spalling tests to measure the free-end velocity and propagating profiles of transmitted wave in concrete bar, which was measured by three strain gauges equidistantly mounted at the loading end of specimen. The free-end velocity was used to estimate the tensile strength by Novikiv-formulation method by presuming linear-elastic materials. Meanwhile, record the movement and deformation of specimen by the ultra-high-speed camera.
(3) Digitize the images captured by the ultra-high-speed camera. The digitized results are then interpreted by DIC method to obtain the displacement field of Lagrangian particles. The Tikhonov regularization method is then applied to reconstruct the derivatives using the discrete input data of particle displacement, which were further used to obtain a series of stress–strain curves using the wave propagation method.

## 3. Results and Discussions

In this section, we report the simulation results which validate the improved “1sv + nv” method for a simplified spalling test. We then present the experimental results obtained by spalling tests and optical techniques. Finally, we analyzed the dynamic constitution behaviors characterized through the application of proposed methods on concrete.

### 3.1. Simulation Results

**Figure 7:**
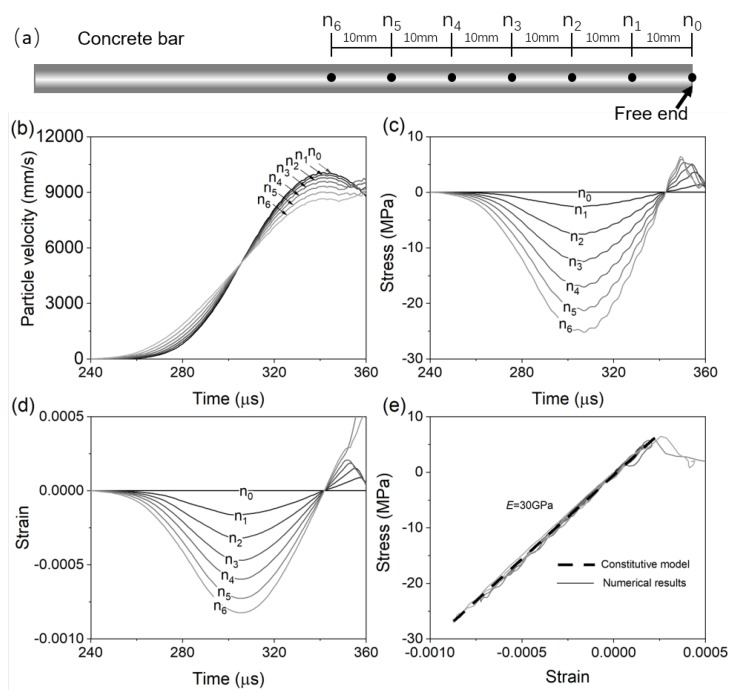
Simulation results for a simplified spalling test model: (**a**) Illustration of seven representative nodes (${}_{0}$ to $n_{6}$), which are selected at 0 to 60 mm away from the free end. The spacing is 10 mm between two neighboring nodes; Simulated Profiles of particle velocity (**b**), stress (**c**), and strain (**d**) for each node; (**e**) Comparing the stress–strain curves obtained by the improved “1sv + nv” method against the constitutive profile defined in the FE model.

Figure 7 shows the simulation results for a simplified spalling test. In Figure 7, we compare the simulated profiles of particle velocity, stress, and strain profiles at seven representative nodes along the concrete bar. These nodes are selected at 0 to 60 mm away from the free end with a spacing of 10 mm (see Figure 7a). In this spalling test, there are actually 60 nodes in total which are located at 0 to 60 mm away from the free end (with a spacing of 1 mm). We thus obtained 60 profiles of particle velocity, stress, and strain from simulation and the improved “1sv + nv” method. In Figure 7, however, only representative nodes with a spacing of 10 mm are selected to illustrate the evolution of velocity, stress, and strain for the purpose of clarity.

Figure 7b shows the particle velocity versus time for seven representative nodes. The particle velocity values at each node are direct output from the FE solver. Figure 7c,d shows each node’s stress and strain profiles, which are calculated by the improved “1sv + nv” method (Equations (1) and (2). Figure 7e compares with the stress–strain profiles determined by the improved “1sv + nv” method with the material constitution relation defined in the FE mode. Figure 7e, the relatively good match in stress–strain profiles validates that the improved “1sv + nv” method can effectively characterize the dynamic constitutive behaviors of concrete in spalling tests.

### 3.2. Experimental Results

In this subsection, we provide an evaluation of the loading-rate effect on the stress propagation in the spalling tests. We then show the results of particle-displacement field interpreted by DIC method, and then present a comparative analysis of spalling cracks and particle-displacement profiles. Finally, we show the results of stress–strain curves obtained by the application of proposed method.

#### 3.2.1. The Effects of Loading Rate on Wave Propagation

Under relatively high load rates, wave propagation can significantly impact the evaluation of constitutive parameters for quasi-brittle materials. Here, we evaluate the effect of loading rate on the wave propagation in concrete.

**Figure 8:**
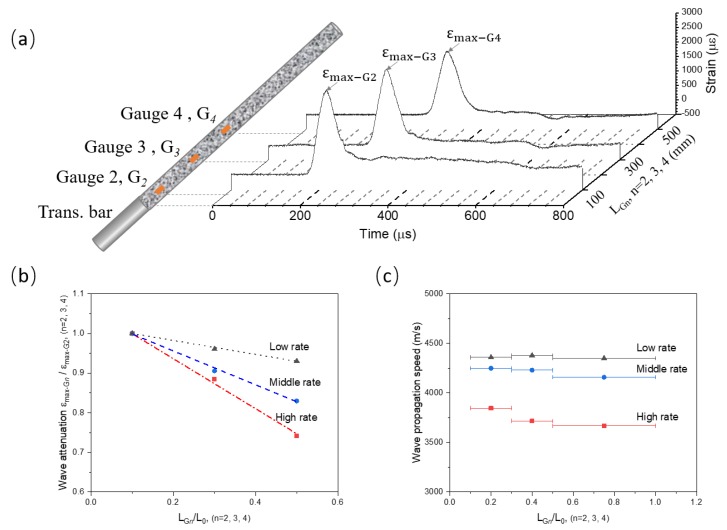
Propagation of stress wave in concrete bar (**a**) is identified by the strain data measured by Gauges 2, 3, and 4. The attenuation (**b**) and propagating speed of (**c**) stress wave are compared with loading rates: Low rate (7 m/s), middle rate (18 m/s), and high rate (33 m/s). $L_{Gn}$ (n=2,3,4) denotes the relative location of three gauges ($G_{2}$, $G_{3}$, and $G_{4}$). $L_{0}$ represents the length of concrete bar.

Figure 8a shows the propagating profiles of stress wave measured by three strain gauges ($G_{2}$, $G_{3}$, and $G_{4}$). These gauges are glued at the surface of concrete bar with locations noted as $L_{Gn}$ (n=2,3,4). Each gauge measures a strain-time curve of local particle, which is further analyzed to evaluate the propagation of stress wave. Below we mainly compare the attenuation and propagating speed of stress wave with three loading rates: Low rate (7 m/s), middle rate (18 m/s), and high rate (33 m/s), where the velocities are the impact velocities of striker.

Figure 8b compares the attenuation of stress wave with loading rates. The attenuation of stress wave is normalized by dividing the peak value of $G_{2}$ curve ($\varepsilon_{max-G2}$). $L_{Gn}$ (n=2,3,4) is normalized by the length of concrete bar ($L_{0}$). In Figure 8b, the results show the attenuation of stress wave significantly decreases with loading rate, suggesting a high sensitivity of wave propagation to loading rate.

Figure 8c compares the propagating speed of stress wave with load rate. The values of propagating speed values averaged for the intervals from G2 to G3, G3 to G4, and G4 to the free end of concrete bar. The results show that the propagating speed of stress wave decreases with loading rate. The propagating speed of stress wave at high loading rate is significantly lower compared with that at low loading rate. However, $C_{0}$ is generally assumed as a constant value for the Novikov-formula (Equation (3). One may thus expect that the assumption of constant $C_{0}$ can lead to significant errors on the estimation of tensile strength of concrete-like materials.

#### 3.2.2. Particle Fields of Displacement, Velocity, Stress and Strain

##### Under Low Loading Rate

**Figure 9:**
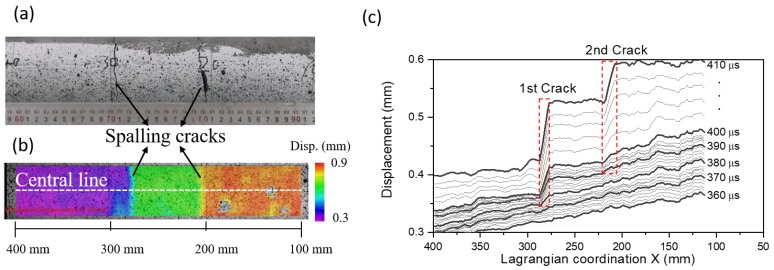
Results of particle displacements and crack profiles in the spalling test at a relatively low loading rate (7 m/s): (**a**) Two spalling cracks are identified in the specimen after the test; (**b**) The particle-displacement field shows two discontinuities at the locations of spalling cracks. The particle-displacement field is characterized by the ultra-high-speed imaging and DIC method. The color scale represents the values of particle displacement; and (**c**) comparing the particle-displacement profiles during t= 360 to 410 μs shows that the left crack initiates earlier than the right one in (**a**).

Figure 9a shows the final state of specimen after the spalling test with loading rate of 7 m/s. In Figure 9a, we observe two spalling cracks in the specimen. Once after the initiation of first spalling crack, the stress state of wave with respect to X becomes uncertain. It may thus violate the assumption of one-dimensional wave propagation in our proposed “1sv + nv” method after the spalling failure. The improved “1sv + nv” method is thus only applicable to X ranging from the free end to the location of first spalling crack. It thus requires identification of the relative sequence of two spalling cracks, and to determine their corresponding locations where they initially develop.

Figure 9b shows the particle-displacement field interpreted by the DIC method after the spalling test. A total of 173 Lagrangian particles are identified in the full-field measurement. These particles are distributed along the central line for X varying from 110 mm to 400 mm of concrete bar. We set X = 0 at the free end. The DIC measured numbers of Lagrangian particles in tests (up to 2 particles/mm) are larger than the node numbers of FE model (1 node/mm) in unit dimension. The spatial resolution of DIC full-field measurements is thus expected to be high enough to reconstruct the derivatives of $\frac{\partial u}{\partial t}$ and $\frac{\partial^{2}u}{\partial t^{2}}$ in our improved “1sv + nv” method.

In Figure 9b, two spalling cracks can also be identified by the discontinuities in the particle-displacement field. We then examine the evolution of full-field particle displacement with time to identify the relative sequence and initiating positions of these two cracks. Figure 9c plots the particle-displacement profiles during the test period of 360 to 410 μs. As shown in Figure 9c, the first spalling crack initiates from X = 286 mm at around 280 μs, whereas the second one initiates from X = 220 mm at about 290 μs. Our proposed “1sv + nv” method is thus only applicable to these Lagrangian particles between the free end and the location of first spalling crack (*X* = 286 mm).

##### Under High Loading Rate

**Figure 10:**
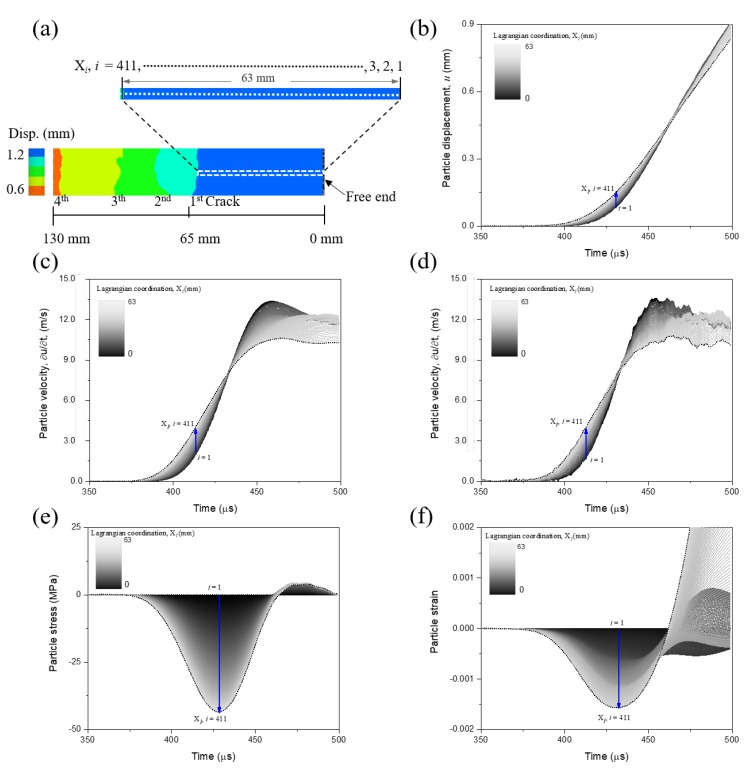
Results of particle displacement, velocity, stress and strain for the spalling test at relatively high loading rate (33 m/s): (**a**) Particle-displacement field characterized by DIC. Four spalling cracks are identified by the discontinuities in particle displacement. A total of 411 particles were identified between the first crack (X= 63 mm) and free end; (**b**) Profiles of displacement versus time for the particles located between X= 0 to 63 mm; (**c**) Particle velocity (X= 0 to 63 mm) calculated by the derivative of particle displacement, which is further reconstructed by the Tikhonov regularization method; (**d**) Particle velocity (X= 0 to 63 mm) obtained from the derivative of particle displacement, which is further reconstructed by the direct smoothing method; (**e**,**f**) Profiles of particle stress and strain versus time for the particles ranging from X= 0 to 63 mm by the improved “1sv + nv” method.

Figure 10a shows the particle-displacement field for the spalling test at a relatively high loading rate (33 m/s). The particle-displacement field is also characterized by DIC method. As shown in Figure 10a, we observe two more spalling cracks with a closer spacing in the high loading-rate test than that identified in the low loading rate (see Figure 9b).

Similarly, we determine the relative sequence of these spalling cracks and corresponding locations where they initially develop for the spalling test at high load rate. The first crack approximately initiates at *X* = 63 mm from the free end of concrete bar. In this test, a total of 411 Lagrangian particles are located between *X* = 0 and *X* = 63 mm. We thus obtain 411 stress–strain curves using the proposed “1sv + nv” method through this high loading-rate test.

The DIC analysis can only provide the particle-displacement fields. However, particle velocity is required to determine the stress and strain profiles using Equations (1) and (2). As shown in Figure 10b, the results show a relatively steep profiles of displacement against time. Small errors in the displacement may thus lead to significant errors in particle velocity. We thus carefully reconstruct the derivatives of particle velocities by the Tikhonov regularization method.

Figure 10c,d show the particle velocity calculated by the Tikhonov regularization method and direct moving-average smoothing method, respectively. The profiles of article velocity reconstructed by Tikhonov regularization method are relatively less noisy compared with that obtained by the smoothing method. As shown in Figure 10e,f, the stress and strain curves against time for particles within X= 0 to 63 mm in the spalling test. The stress and strain data were calculated using Equations (6) and (7) based on the regularized particle velocity.

#### 3.2.3. Dynamic Constitutive Behaviors

**Figure 11:**
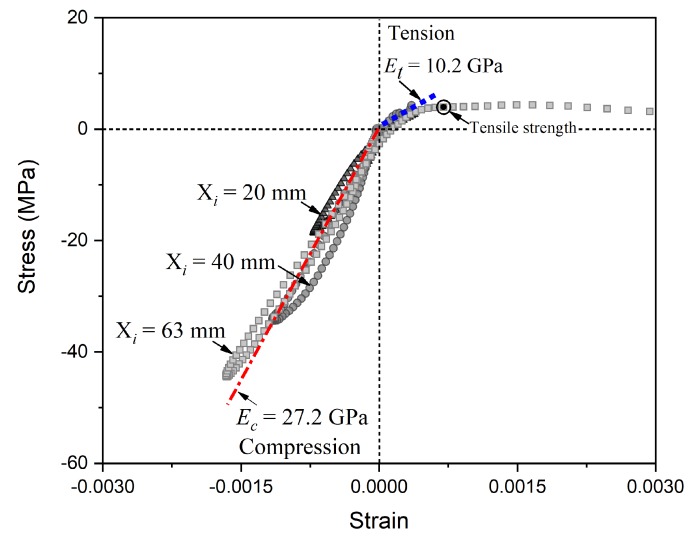
The non-linear behaviors of stress–strain curves for the compression and tension stages including the non-linear increasing elasticity, the different elastic modulus of tension and compression, and non-linear hysteresis loop, for the spalling test under the loading rate of 33 m/s.

In Figure 11, we plot three stress–strain curves of particles at X= 20 mm, 40 mm, and 63 mm for the spalling test under the loading rate of 33 m/s. The stress and strain data were determined by the applying improved “1sv + nv” method on the results in Figure 10. In Figure 11, the stress–strain curves can be divided into the compressive and tensile stages. In Figure 11, we observe a non-linear hysteresis loop in the unloading path of compressive stage, suggesting the visco-elastic behavior in the concrete under the high strain-rate loading condition. In Figure 11, we also observe non-linear behaviors in the stress–strain curves during the tension stage. The results show that a non-linear increasing trend in tensile stress before it reaching to peak value in the stress–strain curve for X= 63 mm, which is located at the position of crack initiation.

In Figure 11, we compare the Young’s modulus (*E*) under compressive and tensile stages for the dynamic tests. The Young’s modulus is represented by the average slope of stress–strain curves where stress varies from 0 to the maximum value during each stage. The results show an asymmetric behavior in the Young’s modulus during compressive and tensile stages. The Young’s modulus is about 10.2 GPa during tensile stage, while it is about 27.2 GPa during compressive stage. Similar asymmetric behavior of *E* during compressive and tensile stages was also reported by Forquin and Erzar [42]. Additionally, the improved Lagrangian-inverse method provides an alternative mean to obtain *E* during tensile stage, which is challenging to be determined in previous studies.

As listed in Table 2, we further compare dynamic the constitutive parameters for the spalling tests under different strain rate. It is challenging to maintain a constant strain rate or stress rate due to the dynamic superposition of stress waves. To obtain a constant strain rate at failure, the shape of loading wave should be carefully designed. In this study, we employed several pure copper rings to obtain triangle pulses with different up-rising and down-rising edges.

Table 2 summarizes the results of strain rate at failure ($\dot{\gamma_{f}}$), maximum tensile stress ($\sigma_{fmax}$), tensile stress calculated by the pull-back velocity ($\sigma_{fdyn}$) using Novikov formulation (Equation (3) for the tests with different loading rates. $\dot{\gamma_{f}}$ and $\sigma_{fmax}$ are determined by the proposed method. The Young’s modulus is set as quasi-static compressive one, 32 GPa, for calculating $\sigma_{fdyn}$. In Table 2, the results show that $\sigma_{fdyn}$ is larger than $\sigma_{fmax}$ under the same strain rate. Also, $\sigma_{fdyn}$ increases faster than $\sigma_{fmax}$ with increasing strain rates.

## 4. Discussions

There are three main experimental methods to conduct the dynamic tensile tests on concrete-like materials: The direct tensile method, the dynamic Brazilian splitting test, and the spalling test. Although the direct tensile method is the most ideally way to get the dynamic uniaxial tensile behaviors of concrete-like materials, rarely effective instruments were reported to successfully obtain the dynamic uniaxial stress–strain curves under high-strain-rate loading. For the dynamic Brazilian splitting, only a tensile strength can be got but with a biaxial stress state. The classical spalling tests can get a uniaxial tensile strength but with an elastic presumption on materials. In this article, our improved Lagrangian-inverse method used in spalling tests provides a new alternative to obtain not only the dynamic uniaxial tensile properties of concrete but also the dynamic uniaxial compressive properties without any presumption on the constitution.

As the experimental results, the concrete shows significant non-linear constitutive behaviors under high-strain-rate conditions. The non-linear constitutive behaviors may suggest that the assumption of the linear-elastic constitution of concrete may be invalid for the concrete-like materials. This is supported by previous studies which also report similar non-linear behaviors in concrete-like materials [33,52,53]. One may expect that these non-liner behaviors contribute to the significant reduction in the attenuation and propagating speed of stress wave under high loading-rate conditions.

The non-liner constitutive behaviors can be possibly explained by the local heterogeneity of concrete materials. Ideally, the stress wave is expected to propagate in one dimension through the concrete bar. However, the one-dimension wave propagation can be affected by transmission and reflection which occur at the interface of cement, fine aggregates and coarse aggregates. Also, similarly, the wave propagation can also be impacted by the local bubbles, cracks, and faults in concrete at macro- to micro-scale. Once after the loading of impact pulse, these macro- and micro-scale structures in concrete may inevitably lead to unrecoverable deformation, which further leading to the macro-scale non-liner constitutive behaviors.

The non-linear behavior at the tensile stage is more pronounced than that at the compressive stage, leading to more evident asymmetric behavior of *E* during compressive and tensile stages. Also, the non-linear behavior can significantly impact the strain-rate effect of tensile strength. As listed in Table 2, the estimated tensile strength ($\sigma_{fmax}$) at the strain rate of 120 s${}^{-1}$ is 1.45 times larger than that under quasi-static condition. However, $\sigma_{fdyn}$ at the strain rate of 120 s${}^{-1}$ is about 3.4 times larger than the tensile strength under quasi-static condition. $\sigma_{fdyn}$ was estimated by presuming linear-elastic constitution, whereas the testing results clearly show non-linear constitution behavior. One may thus expect that presuming linear-elastic constitution would significantly overestimate the strain-rate effect for the concrete-like materials.

## 5. Summary and Conclusions

In this study, we evaluated the dynamic constitutive behaviors of ordinary concrete at high strain rates through combing Lagrangian-inverse analysis method with optical techniques. We proposed an improved “1sv + nv” method, and then validated it against finite-element simulation. We further applied the proposed method on the spalling tests to evaluate the constitutive behaviors of ordinary concrete at high strain rates. The key conclusions can be summarized as follows:

(1) The relatively good match of the stress–strain curves against simulation results validates the improved “1sv + nv” method.
(2) Comparative analysis of the spalling test results suggests that wave propagation in concrete can be significantly affected by loading rate. increasing loading rates can significantly reduces the attenuation and propagating speed of stress wave in concrete.
(3) The Tikhonov regularization method is proved to be more effectively reduce the noise in reconstructing particle velocity compared with moving-average smoothing method.
(4) Non-linear constitutive behaviors are identified in the stress–strain curves obtained by applying the improved “1sv + nv” method to the spalling test and DIC results. The non-liner constitutive behaviors can be possibly explained by the local heterogeneity of concrete materials.
(5) The DIC method inevitably associates with periodic oscillations due to the “breathing Effect” of ultra-high-speed camera. The accuracy in particle displacement might be slightly affected by the treatment of noise caused by periodic oscillations. Additionally, the proposed method provides an alternative mean to access the dynamic stress–strain curves for concrete. Future works are recommended to investigate the applicability of the proposed method on brittle and quasi-brittle materials such as geomaterials, cement-based materials, ceramics, and glasses.

## Figures and Tables

**Table 1:** Quasi-static mechanical properties of concrete.

	Density	Compressive Strength	Splitting Tensile Strength	Compressive Young’s Modulus
Concrete	kg/m${}^{3}$	MPa	MPa	GPa
	2400	60	4.6	32

**Table 2:** Strain rates and tensile strengths.

Loading Rates	Numb.	$V_{I}$ (m/s)	${\dot{\gamma }}_{f}$ (1/s)	$\sigma_{\mathrm{fmax}}$ (MPa)	$\sigma_{\mathrm{fdyn}}$ (MPa)
Quasi-static splitting	01	−	$10^{-4}$	4.60	−
Low rates	01	7.00	11.4	4.31	6.74
	02	7.00	16.7	5.17	6.59
	03	7.00	18.1	4.46	7.50
Middle rates	01	18.0	43.8	5.49	11.7
	02	18.0	47.5	4.35	12.2
	03	18.0	55.3	5.87	12.1
High rates	01	33.0	87.9	7.14	13.7
	02	33.0	96.5	4.82	14.5
	03	33.0	120	6.70	15.6

## Abbreviations

The following abbreviations are used in this manuscript:

1D	One-dimensional
DIC	Digital image correlation
FE	Finite-element method
SHPB	Split Hopkinson pressure bar
SGF	Sativetzky–Golay filtering method
TRW	Tikhonov regularization method
VFM	Virtual field method
WPT	Wave propagation technique
*B*	The perturbation amplitude value
$C_{0}$	The speed of elastic pulse in concrete
$E_{c}$	The compressive Young’s modulus
$E_{t}$	The tensile Young’s modulus
*i*	The space increment of *X* in Lagrangian coordination
*j*	The time increment of *t*
*k*	The order of derivative
*N* or *n*	The number of the data
*t*	Time
*v*	The particle velocity
$V_{0}$	The particle velocity of free end
$V_{I}$	The impact velocity of strike bar
*X*	The spatial variable in Lagrangian coordination
α	The regularization parameter
$\Delta V_{pb}$	The “Pull-back” velocity
δ	The error
ε	The particle strain
$\rho_{0}$	The density of concrete
σ	The particle stress
$\sigma_{fdyn}$	The tensile strength calculated by the Novikov-formula
$\sigma_{fmax}$	The tensile strength calculated by the improved method

## Appendix A. Tikhonov Regularization Method

In this Appendix, we briefly review the Tikhonov regularization method (TRM). We then present a comparative analysis through one example to evaluate the TRM.

### Appendix A.1. A Brief Review

Consider a total number of n + 1 of ${\tilde{y}}_{i}$ data uniformly sampled with an error of δ. To obtain the k-order derivative, a function *f* is introduced to approximate the data ${\tilde{y}}_{i}$. *f* can be obtained by minimizing the function described by

(A1) $$\Phi \left(f\right)=\frac{1}{n-1}\sum_{j=1}^{n-1}{\left({\tilde{y}}_{j}-f\left(x_{j}\right)\right)}^{2}+\alpha {∥f^{\left(k\right)}∥}_{L^{2}\left(0,1\right)}^{2}$$

where n+1 is the total number of the data; $x_{j}$ is the sample time where $\left\{0=x_{0}<x_{1}<\cdots <x_{n}=1\right\}$; α is a regularization parameter which is related to δ; and *f* is the function constrained by $f\left(0\right)=\tilde{y}\left(0\right)$ and $f\left(1\right)=\tilde{y}\left(1\right)$.

The paper proves that the solution for *f* is unique. *f* is can be expressed by piece-wise polynomial as follows:

(A2) $$f_{*}\left(x\right)=c_{j,1}+c_{j,2}x+\cdots +c_{j,2k}x^{2k-1},x\in \left(x_{j},x_{j+1}\right),j=0,1\cdots n-1$$

$c_{j,1}$ to $c_{j,2k}$ can be obtained by solving the following linear equations:

(A3) $$\left\{\begin{array}{c} {f_{*}}^{\left(i\right)}\left(x_{j}+\right)-{f_{*}}^{\left(i\right)}\left(x_{j}-\right)=0,i=0,1,\cdots 2k-2,j=1,2,\cdots n-1\\ {f_{*}}^{\left(2k-1\right)}\left(x_{j}+\right)-{f_{*}}^{\left(2k-1\right)}\left(x_{j}-\right)=\frac{{\tilde{y}}_{j}-f_{*}\left(x_{j}\right)}{\alpha {\left(-1\right)}^{k}\left(n-1\right)},j=1,2,\cdots n-1\\ {f_{*}}^{\left(k+j\right)}\left(1\right)=0,j=1,2,\cdots k-2\\ {f_{*}}^{\left(k+j\right)}\left(0\right)=0,j=1,2,\cdots k-2\\ f_{*}\left(0\right)=y\left(0\right),f_{*}\left(1\right)=y\left(1\right) \end{array}\right.$$

The regularization parameter, α, in Equation (A1) can be determined by

(A4) $$\alpha =\delta^{2}$$

### Appendix A.2. Comparative Analysis

Given a function of y described by

(A5) $$y=0.5\times \left(1-\cos(x)\right)+B\times random\left(-1,1\right),x\in \left[0,\pi \right]$$

where *B* is the perturbation amplitude value; and random(−1,1) generates random values between −1 and 1.

In Figure A1, we compare the precise values of *y* data with that calculated by Equation (A5). B is set to 0.04, which is twice lager than that obtained by DIC analysis. The precise values of *y* are calculated by the function of $y1=0.5\times \left(1-\cos(x)\right)$.

In Figure A2, we compare the first derivative data smoothed by Tikhonov regulation method (TRM) and the Sativetzky–Golay filtering (SGF) method with the function $y_{1}^{\prime }$. We set the window size as 30 and the degree of the Polynomial at 2 in these smoothing methods. The results show a relatively good match between the first derivative data smoothed by TRM and the $y_{1}^{\prime }$ function. Also, comparative analysis suggests that TRM can more effectively filter the first derivative data noise compared with the SGF method.

In Figure A3, we compare the second derivative of *y* data smoothed by Tikhonov regulation method with that calculated by $y_{1}^{\prime \prime }$ function. Figure A3 also plots the absolute error to the right vertical axes. The results show a relatively good match between the smoothed data and $y_{1}^{\prime \prime }$ except for slightly minor error at both ends.
